# Comparative cardiac macroscopic and microscopic study in cats with hyperthyroidism vs. cats with hypertrophic cardiomyopathy

**DOI:** 10.1080/01652176.2023.2234436

**Published:** 2023-07-18

**Authors:** Izabela Janus, Agnieszka Noszczyk-Nowak, Joanna Bubak, Massimiliano Tursi, Cristina Vercelli, Marcin Nowak

**Affiliations:** a Department of Pathology, Wrocław University of Environmental and Life Sciences, Wrocław, Poland; b Department of Internal Diseases with Clinic of Dogs, Cats and Horses, Wrocław University of Environmental and Life Sciences, Wrocław, Poland; c Department of Veterinary Science, University of Turin, Grugliasco, Italy

**Keywords:** Feline hyperthyroidism, cardiac hypertrophy, hypertrophic cardiomyopathy, cardiac remodelling, histopathology

## Abstract

Hyperthyroidism is considered the most common endocrinopathy in middle-aged and old cats. The increased level of thyroid hormones influences many organs, including the heart. Cardiac functional and structural abnormalities in cats with hyperthyroidism have indeed been previously described. Nonetheless, myocardial vasculature has not been subjected to analysis. Also, no comparison with hypertrophic cardiomyopathy has been previously described. Although it has been shown that clinical alterations resolve after the treatment of hyperthyroidism, no detailed data have been published on the cardiac pathological or histopathological image of field cases of hyperthyroid cats that received pharmacological treatment. The aim of this study was to evaluate the cardiac pathological changes in feline hyperthyroidism and to compare them to alterations present in cardiac hypertrophy due to hypertrophic cardiomyopathy in cats. The study was conducted on 40 feline hearts divided into three groups: 17 hearts from cats suffering from hyperthyroidism, 13 hearts from cats suffering from idiopathic hypertrophic cardiomyopathy and 10 hearts from cats without cardiac or thyroid disease. A detailed pathological and histopathological examination was performed. Cats with hyperthyroidism showed no ventricular wall hypertrophy in contrast to cats with hypertrophic cardiomyopathy. Nonetheless, histological alterations were similarly advanced in both diseases. Moreover, in hyperthyroid cats more prominent vascular alterations were noted. In contrast to hypertrophic cardiomyopathy, the histological changes in hyperthyroid cats involved all ventricular walls and not mainly the left ventricle. Our study showed that despite normal cardiac wall thickness, cats with hyperthyroidism show severe structural changes in the myocardium.

## Introduction

The first reports on feline hyperthyroidism (FHT) were published in 1979–1980 (Holzworth et al. 1980; Mooney 2002; Peterson 2014; Carney et al. 2016). Since then, not only our knowledge on the disease pathogenesis, consequences and treatment has developed, but also the disease occurrence and diagnosis have increased (Mooney 2002; Gunn-Moore 2005; Peterson 2014; Carney et al. 2016). FHT is diagnosed in up to 11.4% of older cats around the world and is considered the most common endocrine disorder in middle-aged or older cats without sex predisposition (Mooney 2002; Gunn-Moore 2005; Stephens et al. 2014; Carney et al. 2016; McLean et al. 2017).

With the growing awareness of the disease, new treatment methods were established: from treatment using methimazole or carbimazole to radioactive iodine (^131^I) therapy, iodine-restricted food or surgical thyroidectomy (Gunn-Moore 2005; Carney et al. 2016).

The causes of the disease in cats are still not evident: genetic, immunologic, hormonal, dietary and environmental factors can play a role in the development of FHT (Mooney 2002; Gunn-Moore 2005; Carney et al. 2016).

The clinical presentation of a hyperthyroid cat can vary due to the broad role of thyroid hormones in the organism (Gunn-Moore 2005; Carney et al. 2016). Several organs can be affected with resultant behavioural alterations, cardiovascular abnormalities, hypertension, malnutrition and intestinal malabsorption, cachexia, nervous alterations, and chronic renal failure with decreased urine-concentration ability (Graves 2011). One of the comorbidities present in FHT is heart disease, which can eventually lead to congestive heart failure. The cardiac effect is complex and can be associated with high-output state resulting from the need to sustain increased tissue perfusion to meet the requirements of elevated ­tissue metabolism, or from changes in under- or overexpression of various genes responsible for ­cardiomyocyte structure and function and up-regulation on beta-1 adrenoceptors in the cell membrane of ­cardiomyocytes, resulting in a positive inotropic, ­dromotropic and chronotropic effect (Gunn-Moore 2005; Vargas-Uricoechea and Sierra-Torres 2014; Osuna et al. 2017). Thyroid hormones can also have a direct effect on cardiac muscles, although the mechanism is not precisely described (Gunn-Moore 2005). The changes in heart function may resolve after achieving euthyroid state (Carney et al. 2016; Kittleson and Côté 2021), but experimental studies conducted on mice showed that older animals have a lower recovery potential (Hübner et al. 2015). Due to its impact on cardiac function, hyperthyroidism is among the disorders listed in differential diagnosis of hypertrophic cardiomyopathy (HCM) in the case of mild to moderate left ventricular wall thickening (Kittleson and Côté 2021).

FHT shows similarities with human toxic nodular goitre, therefore is considered to be a model for this disease (Peterson 2014). In humans, elevated thyroid serum hormone levels cause measurable alterations in various cardiac parameters, including an increase in resting heart rate, myocardial contractility and left ventricular muscle mass, and a predisposition to supraventricular arrhythmias (Fadel et al. 2000; Biondi et al. 2005; Biondi and Kahaly 2010; Biondi 2021). The cardiac function alterations can also be present in subclinical forms of hyperthyroidism (Biondi 2021). Although the genetic and molecular mechanisms of the disease resulting in the abovementioned cardiac alterations have been studied in humans and laboratory animals (Fadel et al. 2000; Bektur Aykanat et al. 2021), the coronary vasculature alterations have not been a subject of detailed analysis.

Up to date, the cardiac pathological changes have been studied in detail in hyperthyroid animals (including cats and laboratory animals) with or without treatment (Liu et al. 1984; Hoey et al. 1991; Noszczyk-Nowak 2007; Noszczyk-Nowak et al. 2007; Freitas et al. 2013; Hübner et al. 2015; Bektur Aykanat et al. 2021). The majority of papers refer to experimental cases (Hoey et al. 1991; Noszczyk-Nowak 2007; Noszczyk-Nowak et al. 2007; Freitas et al. 2013; Hübner et al. 2015; Bektur Aykanat et al. 2021).

The aims of this study were: (1) to describe the histopathological characteristics of hearts obtained from cats with hyperthyroidism; (2) to compare the pathological image of cardiac changes in FHT and HCM.

## Materials and methods

The study was conducted on hearts obtained during necropsy from 40 cats subjected to necropsy in the Department of Pathology at the Wroclaw University of Environmental and Life Sciences and the Department of Veterinary Sciences at the University of Turin. All subjects were owned-cats, and informed consents were signed prior to necropsy to allow the procedure and sample collection. All animals were euthanised with an intravenous injection of pentobarbital solution. According to the national law, studies conducted on tissue samples obtained from necropsy do not require approval from the local ­ethics committee.

Based on clinical information, animals were divided into hyperthyroidism (FHT group; *n* = 17), hypertrophic cardiomyopathy (HCM group; *n* = 13) and control (*n* = 10) groups. Prior to death and necropsy, animals from the FHT and HCM groups were diagnosed and treated in accordance with the appropriate guidelines (Carney et al. 2016; Luis Fuentes et al. 2020; Supplementary Material). The diagnosis of cats in the FHT group was based on clinical symptoms and thyroid hormone measurements. The animals received pharmacological treatment. All cats in the HCM group were diagnosed on the basis of clinical symptoms and echocardiographic examination. Other conditions resulting in cardiac hypertrophy (e.g. kidney failure, systemic hypertension, hyperthyroidism) were excluded in this group. The control group consisted of animals with a known clinical history without cardiac, thyroid or renal diseases and systemic hypertension. Their euthanasia was a result of severe trauma (road traffic injuries or severe dog bite).

**Figure 1.**
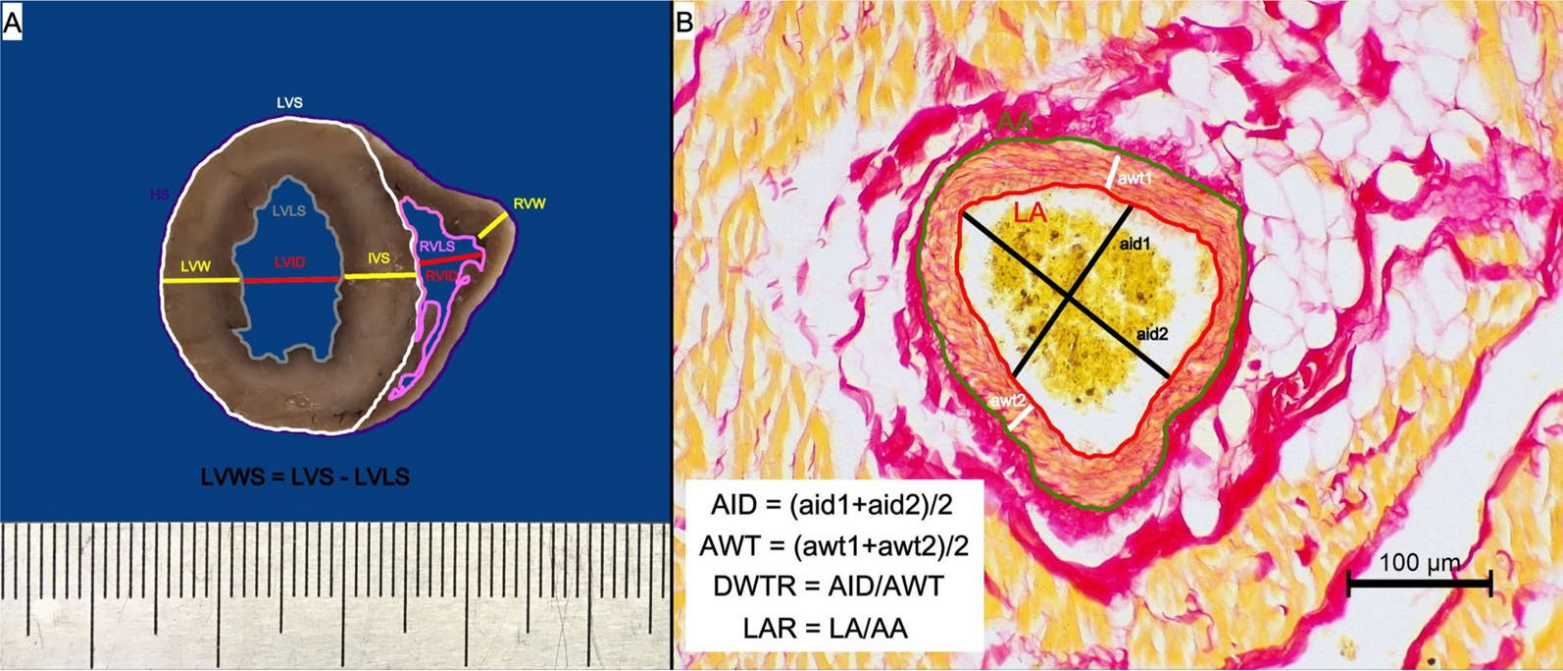
The methods of gross pathological and histological morphometrical analysis. (A) The methodology of the gross measurements of the studied hearts that were conducted on the transverse section of the ventricular wall at the level of the upper third of the ventricle (below the valvular ring). (B) The methodology of the histological morphometric measurements of intramyocardial coronary arteries as proposed by Falk et al. (2006); picro sirius red stain, 200× magnification. AA: arterial area; aid: measured arterial internal diameter; AID: calculated arterial internal diameter; awt: measured arterial wall thickness; AWT: calculated arterial wall thickness; DWTR: arterial diameter-to-wall thickness ratio; HS: heart surface; IVS: interventricular septum; LAR: arterial lumen-to-area ratio; LVID: left ventricular internal diameter; LVLS: left ventricular lumen surface; LVS: left ventricular surface; LVW: left ventricular wall; LVWS: left ventricular wall surface; RVID: right ventricular internal diameter; RVLS: right ventricular lumen surface; RVW: right ventricular wall.

After the necropsy, the hearts were fixed for 24 h in 7% buffered formalin and underwent detailed pathomorphological examination. Measurements were taken during the external examination: the height (largest longitudinal diameter) and width (largest transverse diameter) of: the whole heart, left atrial appendage and right atrial appendage. Subsequently, each heart was sectioned transversely at the level of the upper third ventricular height (below the atrio-ventricular valvular ring) and the thickness of ventricular walls and diameter of ventricular lumen were measured as presented in Figure 1. All the measurements were taken using a manual calliper with an accuracy of 0.1 mm. Photographs of the external surface and of the cutting sections of each heart were taken using a Nikon D7000 camera (Nikon Europe B.V., Poland). In the photographs of the transverse ventricular section, further measurements were taken: the heart surface, left ventricular total surface, left ventricular lumen, left ventricular wall surface and right ventricular lumen (presented in Figure 1A).

After gross examination, specimens of the left ventricular wall, right ventricular wall and interventricular septum at transverse section (at the level of the upper third ventricular height) and longitudinal section (at the level of cardiac apex) were obtained from each heart. The specimens were embedded in paraffin blocks and cut into 6 μm sections. The specimens were stained using haematoxylin-eosin (HE) and picro sirius red stains and underwent detailed microscopic examination.

The level of cardiomyocyte degeneration (based on loss of striation and changes in cardiomyocyte structure) and myocardial inflammatory infiltration was evaluated in the left ventricular wall, right ventricular wall and interventricular septum specimens using a semi-quantitative method based on a grading system used previously in dogs (Janus et al. 2016; Table 1). The level of myocardial disarray was evaluated in the left ventricular wall specimens using a semi-quantitative method modified after Biasato et al. (2015), as presented in Table 1. The evaluation was performed using an Olympus CX41 microscope (Olympus, Japan).

**Table 1.** The method of semi-quantitative histological evaluation of cardiomyocyte degeneration, myocardial inflammatory infiltration and myocardial disarray in the ventricular specimens of the examined animals based on Janus et al. (2016) and Biasato et al. (2015).

Score/feature	0	1	2	3
Cardiomyocyte degeneration	Normal myocardium	Subtle cardiomyocyte changes: loss of cross-striation	Moderate cardiomyocyte changes: cardiomyocyte swelling and cytoplasmic degeneration	Severe cardiomyocyte changes: hyperchromatosis and loss of cell structure
Myocardial inflammatory infiltration	No inflammatory cells	Single inflammatory cells dispersed in the myocardium	Multiple inflammatory cells dispersed in the myocardium	Multiple groups of inflammatory cells in the myocardium
Myocardial disarray (only left ventricular wall)	Absent	1–2 areas of disarray in the specimen	3–5 areas of disarray in the specimen	>5 areas of disarray in the specimen

The number of areas of disarray within the left ventricular wall specimen were successively counted and transformed into a semi-quantitative scale as presented in the Table.

Photomicrographs of the left ventricular wall, right ventricular wall and interventricular septum from each specimen stained with HE and picro sirius red stain were taken using a Leica DM500 microscope coupled with a Leica ICC50W camera (Leica Microsystems, KAWA.SKA, Poland) and subjected to further analysis.

Ten view fields with a magnification of 400× obtained from specimens stained with HE served to measure the cardiomyocyte diameter on transverse section. The criteria for the measurement of cardiomyocytes were a circular shape and visibility of the cell nucleus as proposed by Coelho-Filho et al. (2013). Fifty randomly selected cells were measured with no more than 10 measurements per image.

Ten view fields with magnification 100× (total area of 12.3 mm^2^) without blood vessels obtained from specimens stained with picro sirius red stain served to calculate the percentage of fibrosis. All images were transformed into binary images using ImageJ software (LOCI, University of Wisconsin, USA), enabling the differentiation of connective tissue (black) from muscle tissue (white). The proportion of fibrosis was calculated as the percentage of black area per total image area.

Ten intramyocardial coronary arteries photographed at 200× magnification served to calculate the arterial internal diameter, arterial wall thickness, arterial diameter-to-wall thickness ratio (DWTR = arterial internal diameter/arterial wall thickness) and lumen-to-area ratio (LAR = area of arterial lumen/total arterial area) as described previously (Falk et al. 2006) and presented in Figure 1B.

All measurements were taken using ImageJ software (LOCI, University of Wisconsin, USA). The average of the measurements was calculated for each specimen.

The statistical analysis was performed using Statistica 13.3 (StatSoft, Poland) and appropriate tests. The Shapiro-Wilk test was used to test data normality. The difference between the groups was tested with either Kruskal-Wallis analysis with Dunn post-hoc test (for non-parametric data) or one-way ANOVA analysis with post-hoc Bonferroni test (for parametric data). The difference in sex distribution was tested using the chi^2^ independence analysis. The significance level was set at *p* < 0.05.

## Results

The animals’ age, sex, breed and body weight in the study groups are presented in Table 2. The animals were aged from 1 to 20.5 years (median 8 years). The FHT group was significantly older than the HCM group but with no difference from the control group. Also, no age difference was found between the HCM and control groups (Table 2; Kruskal-Wallis analysis). Fifty-eight percent of all animals were males with no differences in sex distribution between the groups (Table 2; chi^2^ independence analysis; *p* > 0.05). The majority of cats (62.5%) were domestic shorthair cats; among other breeds, the most numerous were: Maine Coon (15%) and British shorthair (10%). Domestic longhair, Norwegian, sphynx, Scottish fold and ragdoll breeds were represented by one cat each. The examined groups show no difference in cats’ body weight (Table 2; Kruskal-Wallis analysis; *p* > 0.05).

**Table 2.** The age, sex, body weight and breed of the studied animals.

Group / parameter		Control group *n* = 10	FHT group *n* = 17	HCM group *n* = 13	*p*-values
Age [years] median (min–max)		1.5 (1–17)	14 (4–20.5)	5 (1–15)	^1^*p* > 0.05 ^2^*p* > 0.05 ^3^***p* = 0.01**
Sex (%M:%F)		60%:40%	58.8%:41.2%	53.8%:46.3%	*p* > 0.05
Body weight [kg] median (min–max)		4.65 (3–8)	4.5 (4–8)	4.7 (3.5–7.5)	*p* > 0.05
Breed (n)	DSH	6	14	5	
	DLH	–	1	–	
	BSH	2	–	2	
	MCo	1	1	4	
	Ragdoll	1	–	–	
	Norwegian Forest	–	1	–	
	Sphynx	–	–	1	
	Scottish Fold	–	–	1	

Values presented as median and range for age and body weight (non-parametric data) and ratio of male (M) and female (F) cats for sex; single *p*-value >0.05 presented in parameters without significant differences between the groups; particular *p*-values presented for the comparison of: control and FHT group (^1^), control and HCM group (^2^) and FHT and HCM group (^3^) in parameters with significant differences between the groups. The significance level set at *p* ≤ 0.05. The significant *p*-values marked bold.

BSH: British Shorthair; DLH: domestic longhair; DSH: domestic shorthair; MCo: Maine Coon.

The results of the cardiac gross morphometric analysis are presented in Table 3.

**Table 3.** The results of the pathomorphological examination of the studied hearts.

Group / parameter	Control *n* = 10	FHT *n* = 17	HCM *n* = 13	*p*-values
Heart weight [g] median (min–max)	15 (13–15)	22.5 (18–24)	34.5 (15–45)	^1^*p* > 0.05 **^2^*p* = 0.04** ^3^*p* > 0.05
Heart height [mm] median (min–max)	38.2 (30.3–41.2)	41.3 (36.6–53.6)	50.2 (34.2–63.5)	^1^*p* > 0.05 **^2^*p* < 0.001** ^3^*p* > 0.05
Heart width [mm] mean ± SD	25.8 ± 3.7	32.6 ± 3.3	34.2 ± 5.1	**^1^*p* = 0.003** **^2^*p* < 0.001** ^3^*p* > 0.05
Left atrial appendage height [mm] median (min–max)	10.9 (8.2–13.2)	16.0 (10–29.8)	25.4 (15.3–38)	^1^*p* > 0.05 **^2^*p* < 0.001** ^3^*p* > 0.05
Left atrial appendage width [mm] median (min–max)	10.4 (8.4–11.8)	15.3 (12.5–16.9)	19.9 (12.6–34.2)	**^1^*p* = 0.03** **^2^*p* < 0.001** ^3^*p* > 0.05
Right atrial appendage height [mm] mean ± SD	10.8 ± 1.7	12.9 ± 3.7	15.3 ± 2.9	^1^*p* > 0.05 **^2^*p* = 0.004** ^3^*p* > 0.05
Right atrial appendage width [mm] mean ± SD	9.5 ± 1.2	12.4 ± 3.1	13.7 ± 5.1	*p* > 0.05
LVW [mm] mean ± SD	6.6 ± 0.7	6.9 ± 1.7	10.1 ± 1.6	^1^*p* > 0.05 **^2^*p* < 0.001** ^3^***p* < 0.001**
LVID [mm] median (min–max)	6 (4.9–7.3)	7.3 (4.9–13.2)	9.4 (2.3–13.3)	*p* > 0.05
IVS [mm] mean ± SD	5.7 ± 0.7	6.0 ± 1.5	8.4 ± 1.0	^1^*p* > 0.05 **^2^*p* < 0.001** ^3^***p* < 0.001**
RVID [mm] mean ± SD	7.0 ± 1.7	6.1 ± 3.2	5.5 ± 2.7	*p* > 0.05
RVW [mm] mean ± SD	2.1 ± 0.5	2.9 ± 1.1	4.1 ± 1.4	^1^*p* > 0.05 ^2^**<0.001** ^3^***p* = 0.03**
Heart surface [mm^2^] median (min–max)	572.9 (478.8–745.2)	586.0 (380.0–1069.1)	852.6 (508.5–1326.4)	^1^*p* > 0.05 ^2^***p* = 0.01** ^3^***p* = 0.01**
Left ventricle surface [mm^2^] median (min–max)	373.6 (358.9–471.9)	391.7 (284.8–680.9)	611.9 (392.7–1043.2)	^1^*p* > 0.05 ^2^***p* = 0.001** **^3^*p* = 0.003**
Left ventricular lumen surface [mm^2^] median (min–max)	40.6 (17.1–46.7)	38.0 (11.5–203.4)	42.1 (7.0–166.9)	*p* > 0.05
Left ventricular wall surface [mm^2^] median (min–max)	328.7 (314.9–436.0)	357.8 (236.6–477.5)	582.4 (380.3–986.0)	^1^*p* > 0.05 ^2^***p* = 0.002** **^3^*p* < 0.001**
Right ventricular lumen surface [mm^2^] median (min–max)	69.7 (63.3–98.2)	74.6 (9.4–159.1)	46.1 (20.7–261.2)	*p* > 0.05

IVS: interventricular septum diameter; LVID: left ventricular internal diameter; LVW: left ventricular wall diameter; RVID: right ventricular internal diameter; RVW: right ventricular wall diameter; parametric data presented as mean ± SD; non-parametric data presented as median (min–max); single *p*-value >0.05 presented in parameters without significant differences between the groups; particular *p*-values presented for the comparison of: control and FHT group (^1^), control and HCM group (^2^) and FHT and HCM group (^3^) in parameters with significant differences between the groups; the significance level set at *p* ≤ 0.05; the significant *p*-values marked bold.

The external examination of the hearts revealed that hearts obtained from the FHT group showed increased transverse diameter as compared to the control group (*p* = 0.003; ANOVA analysis), while hearts from the HCM group showed cardiomegaly (both heart longitudinal and transverse diameters were increased; *p* < 0.001, Kruskal-Wallis analysis and *p* < 0.001, ANOVA analysis, respectively) together with cardiac hypertrophy (increased heart weight; *p* = 0.04; Kruskal-Wallis analysis). The atrial appendages height in the FHT group, although higher than in the control group, did not show a significant difference, while cats in the HCM group showed significantly higher values of both left and right appendages height as compared to the control group (*p* < 0.001; Kruskal-Wallis analysis and *p* = 0.004, ANOVA analysis, respectively). Additionally, the left atrial appendage width was increased in the FTH and HCM groups as compared to the controls (*p* = 0.03 and *p* < 0.001, respectively; Kruskal-Wallis analysis) (Table 3).

**Figure 2.**
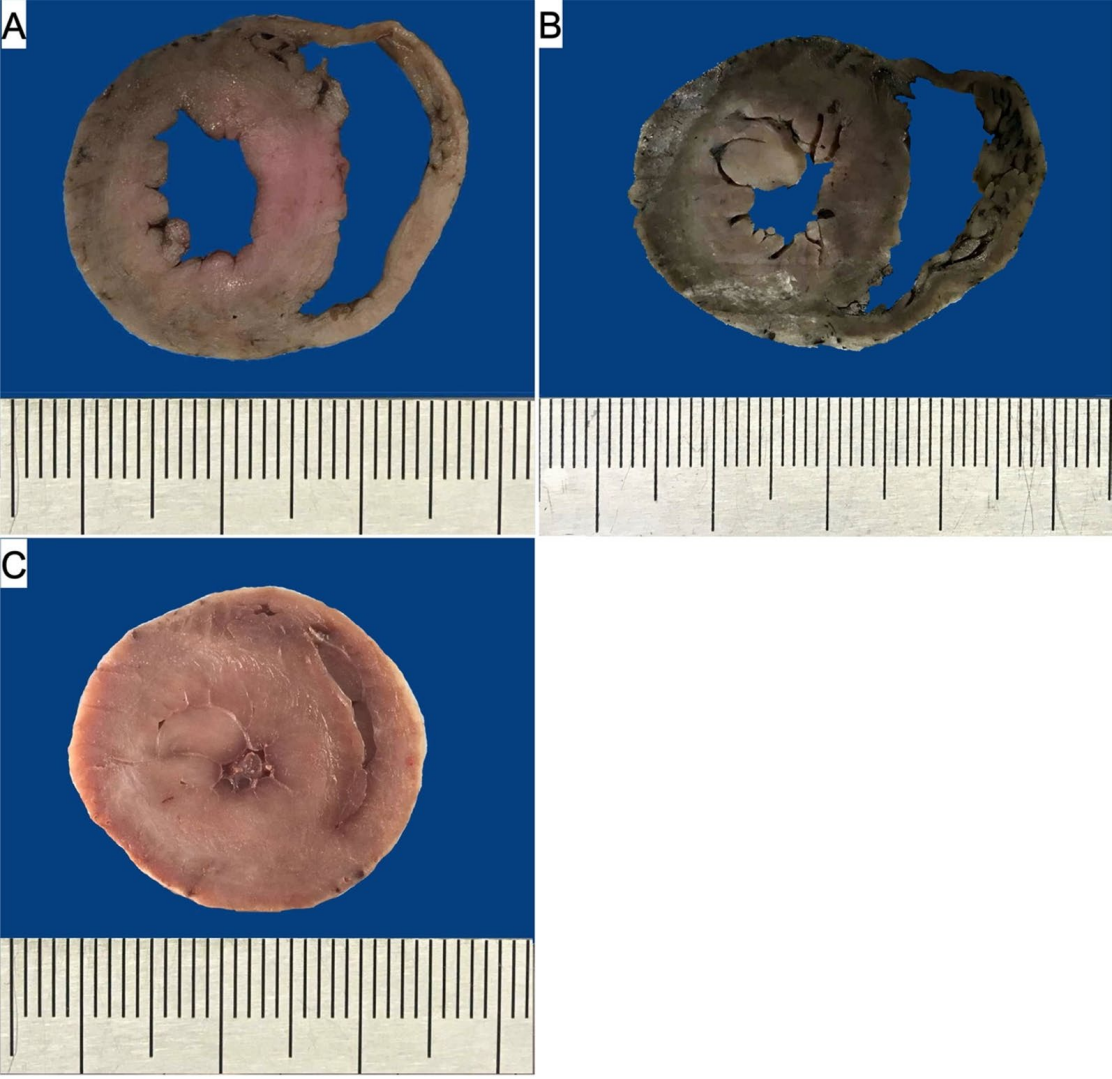
The cross section of the ventricular walls in the control (A), FHT (B) and HCM (C) groups. The patient’s heart weight and their respective body weight: 15 g and 4.3 kg (A), 23 g and 5 kg (B), 39 g and 4 kg (C).

**Figure 3.**
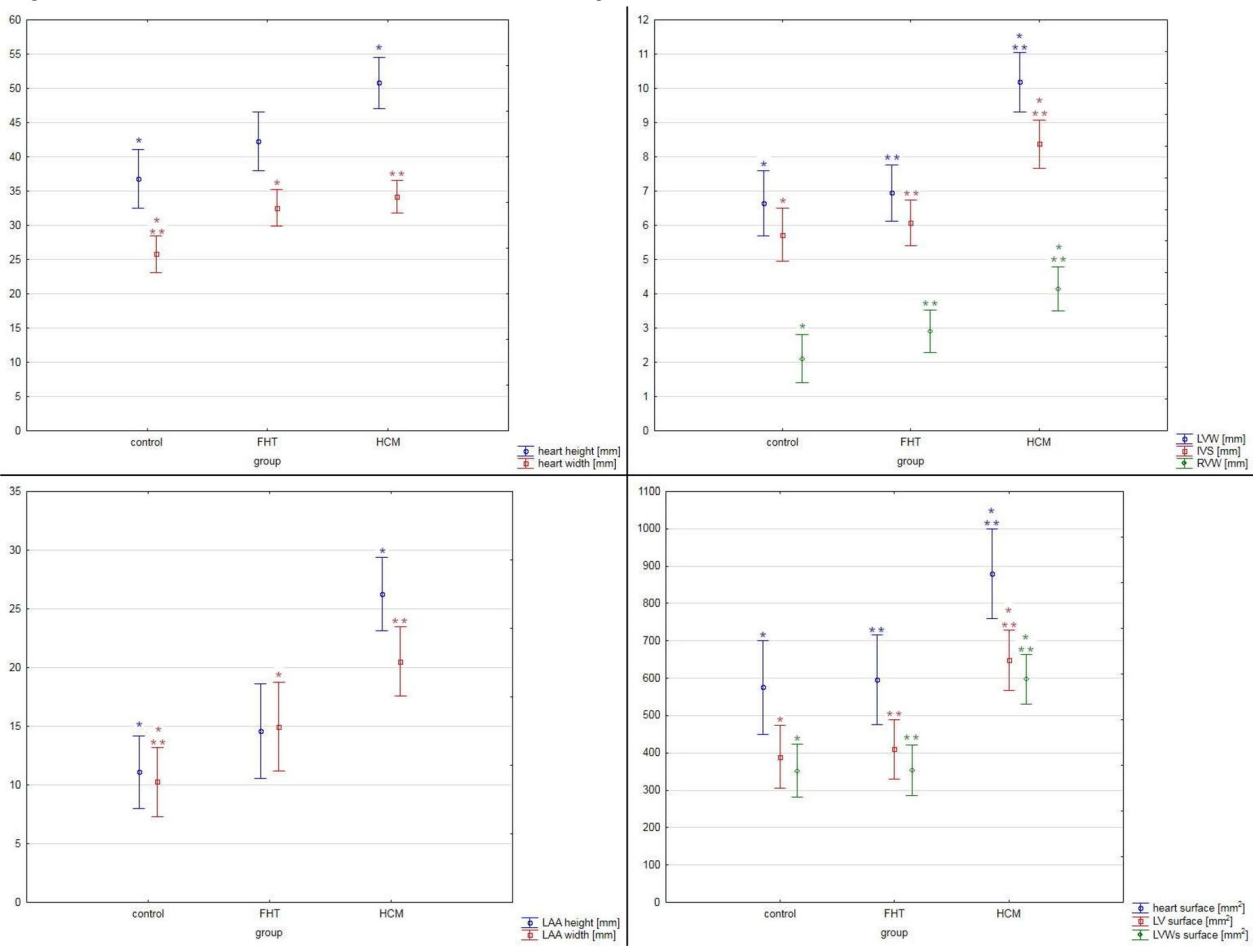
The results of the pathomorphological examination of the studied hearts. The results are presented as mean ± 95% confidence intervals. Values presenting significant differences are marked with asterisk (* or **). IVS: interventricular septum diameter; LAA: left atrial appendage; LV: left ventricle; LVW: left ventricular wall diameter; LVWs: left ventricular walls; RVW: right ventricular wall diameter.

The thickness of the left ventricular free wall, right ventricular free wall and interventricular septum were significantly higher in the HCM group than in the two other groups and no difference was noted between the FHT group and control group (Table 3). Similarly, larger transverse cardiac surface, left ventricular surface and left ventricular wall surface were noted in the HCM group as compared to the FHT and control groups, with no difference between the FHT and control group (Table 3, Figures 2 and 3). The left ventricular hypertrophy was concentric in 62% and eccentric in 38% of cats in the HCM group. In the FHT group, nine cats (53%) showed no left ventricular hypertrophy. In five animals in that group (29%) the hypertrophy was concentric, while in three animals (18%) it was eccentric. Among the hypertrophied hearts in the HCM group, 77% of cases showed symmetrical hypertrophy, and in three cases, the asymmetrical hypertrophy involved the left ventricular free wall. Among the eight hypertrophied hearts in the FHT group, four cases showed symmetrical hypertrophy, three cases – asymmetrical left ventricular free wall hypertrophy and one case – asymmetrical interventricular septum hypertrophy.

The results of the semi-quantitative histopathological analysis are shown in Table 4. Both FHT and HCM groups showed a higher score of cardiomyocyte degeneration (evaluated as described in Table 1) as compared to control cats in all the examined heart walls. The disarray level of the left ventricular wall was higher in the HCM group as compared to the other two groups.

**Table 4.** The results of the semi-quantitative histopathological analysis of the studied hearts.

Group / parameter		Control *n* = 10	FHT *n* = 17	HCM *n* = 13	*p*-values
LVW cardiomyocyte degeneration	median (min–max)	1 (0–1)	3 (1–3)	3 (1–3)	^1^***p* < 0.001** **^2^*p* = 0.001** ^3^*p* > 0.05
	score 0	4	–	–	
	score 1	6	2	1	
	score 2	–	2	5	
	score 3	–	13	7	
IVS cardiomyocyte degeneration	median (min–max)	1 (0–1)	3 (1–3)	3 (1–3)	**^1^*p* < 0.001** **^2^*p* < 0.001** ^3^*p* > 0.05
	score 0	4	–	–	
	score 1	6	2	1	
	score 2	–	3	4	
	score 3	–	12	8	
RVW cardiomyocyte degeneration	median (min–max)	1 (0–1)	3 (0–3)	2 (1–3)	^1^***p* < 0.001** **^2^*p* = 0.02** ^3^*p* > 0.05
	score 0	4	1	–	
	score 1	6	0	3	
	score 2	–	4	7	
	score 3	–	12	3	
LVW myocardial inflammatory infiltration	median (min–max)	0 (0–0)	0 (0–2)	0 (0–1)	*p* > 0.05
	score 0	10	15	11	
	score 1	–	1	2	
	score 2	–	1	–	
	score 3	–	–	–	
IVS myocardial inflammatory infiltration	median (min–max)	0 (0–0)	0 (0–2)	0 (0–0)	*p* > 0.05
	score 0	10	15	13	
	score 1	–	1	–	
	score 2	–	1	–	
	score 3	–	–	–	
RVW myocardial inflammatory infiltration	median (min–max)	0 (0–0)	0 (0–2)	0 (0–1)	*p* > 0.05
	score 0	10	14	11	
	score 1	–	2	2	
	score 2	–	1	–	
	score 3	–	–	–	
LVW myocardial disarray	median (min–max)	0 (0–0)	0 (0–3)	1 (0–3)	^1^*p* > 0.05 **^2^*p* = 0.003** **^3^*p* = 0.01**
	score 0	10	14	2	
	score 1	–	–	6	
	score 2	–	2	3	
	score 3	–	1	2	

IVS: interventricular septum; LVW: left ventricular wall; RVW: right ventricular wall; all data are non-parametric and presented as median (min–max); the number of animals showing score 0–3 are presented in the table; single *p*-value >0.05 presented in parameters without significant differences between the groups; particular *p*-values presented for the comparison of: control and FHT group (^1^), control and HCM group (^2^) and FHT and HCM group (^3^) in parameters with significant differences between the groups; the significance level set at *p* ≤ 0.05; the significant *p*-values marked bold.

In the specimens from the left ventricular wall, a significant cardiomyocyte hypertrophy was noted in the HCM group as compared to the FHT and control groups (*p* = 0.03 and *p* = 0.002, respectively; ANOVA analysis). The FHT group showed a higher left ventricular cardiomyocyte diameter than the control group but did not meet the significance criteria (*p* > 0.05, ANOVA analysis). Simultaneously, interventricular septum cardiomyocytes and right ventricular wall cardiomyocytes showed hypertrophy in both the FHT and HCM group as compared to control cats (*p* < 0.001 for both comparisons in interventricular septum specimens; *p* < 0.001 for FHT–control group comparison and *p* = 0.004 for HCM–control group comparison in the right ventricular wall specimens; ANOVA analysis; Table 5).

**Table 5.** The results of quantitative histopathological examination in the studied groups.

Group / parameter	Control *n* = 10	FHT *n* = 17	HCM *n* = 13	*p*-values
LVW cardiomyocyte diameter mean ± SD	13.6 ± 3.5	16.0 ± 2.9	19.9 ± 5.4	^1^*p* > 0.05 **^2^*p* = 0.002** **^3^*p* = 0.03**
IVS cardiomyocyte diameter mean ± SD	12.4 ± 1.1	17.2 ± 2.7	16.4 ± 1.9	**^1^*p* < 0.001** ^2^***p* < 0.001** ^3^*p* > 0.05
RVW cardiomyocyte diameter mean ± SD	10.7 ± 1.8	16.8 ± 3.5	14.9 ± 2.6	^1^***p* < 0.001** ^2^***p* = 0.004** ^3^*p* > 0.05
LVW fibrosis [%] median (min–max)	6.0 (5.3–7.6)	6.6 (2.5–14.6)	7.1 (2.5–16.9)	*p* > 0.05
IVS fibrosis [%] median (min–max)	4.0 (2.8–5.0)	5.6 (1.4–11.2)	5.6 (1.1–16.2)	*p* > 0.05
RVW fibrosis [%] mean ± SD	7.61 ± 1.8	6.10 ± 2.6	5.85 ± 3.7	*p* > 0.05
LVW arterial diameter-to-wall thickness ratio median (min–max)	4.97 (3.67–5.76)	2.71 (1.44–3.60)	2.70 (2.25–4.73)	**^1^*p* < 0.001** **^2^*p* = 0.001** ^3^*p* > 0.05
IVS arterial diameter-to-wall thickness ratio mean ± SD	4.20 ± 0.36	2.97 ± 0.6	3.39 ± 1.1	^1^***p* = 0.001** ^2^*p* > 0.05 ^3^*p* > 0.05
RVW arterial diameter-to-wall thickness ratio mean ± SD	4.40 ± 0.45	3.37 ± 1.4	3.54 ± 1.2	*p* > 0.05
LVW arterial lumen-to-area ratio median (min–max)	0.39 (0.31–0.41)	0.22 (0.13–0.29)	0.25 (0.19–0.39)	^1^***p* < 0.001** **^2^*p* = 0.01** ^3^*p* > 0.05
IVS arterial lumen-to-area ratio mean ± SD	0.34 ± 0.04	0.24 ± 0.06	0.28 ± 0.06	^1^***p* < 0.001** **^2^*p* = 0.03** ^3^*p* > 0.05
RVW arterial lumen-to-area ratio mean ± SD	0.38 ± 0.04	0.30 ± 0.09	0.35 ± 0.09	^1^***p* = 0.02** ^2^*p* > 0.05 ^3^*p* > 0.05

IVS: interventricular septum; LVW: left ventricular wall; RVW: right ventricular wall; parametric data presented as mean ± SD; non-parametric data presented as median (min–max); single *p*-value >0.05 presented in parameters without significant differences between the groups; particular *p*-values presented for the comparison of: control and FHT group (^1^), control and HCM group (^2^) and FHT and HCM group (^3^) in parameters with significant differences between the groups; the significance level set at *p* ≤ 0.05; the significant *p*-values marked bold.

**Figure 4.**
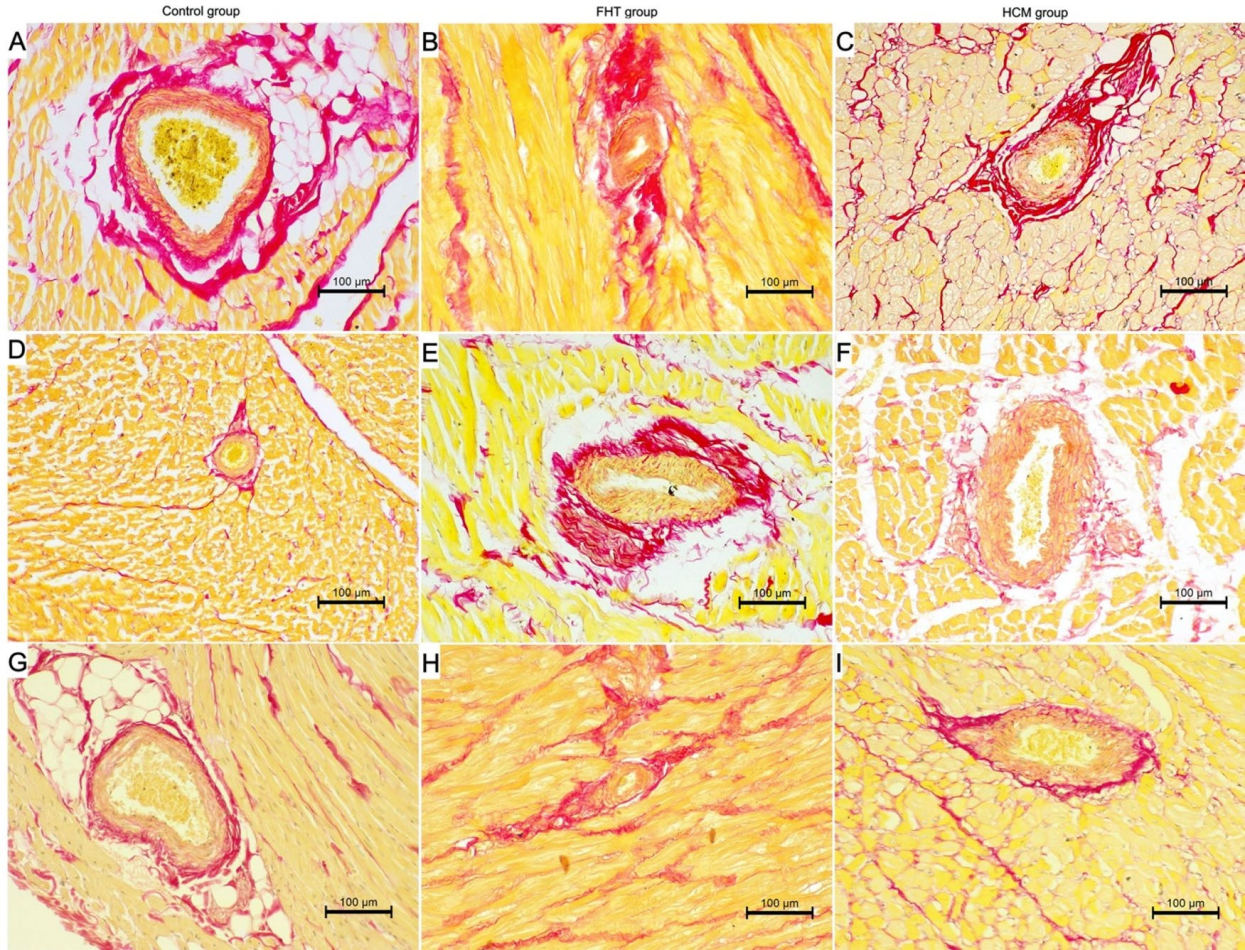
The histological image of the intramural arteries in the examined cats. Picro sirius red stain; 200× magnification. (A, D, G) Control group; (B, E, H) FHT group; (C, F, I) HCM group; (A–C) left ventricular free wall; (D–F) interventricular septum; (G–I) right ventricular free wall. IVS: interventricular septum; LVW: left ventricular free wall; RVW: right ventricular free wall.

**Figure 5.**
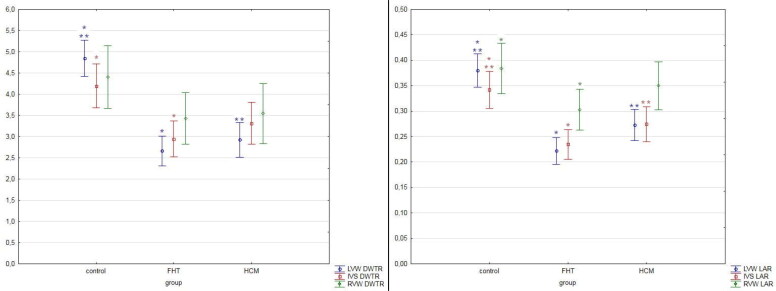
The results of the measurements of coronary arteries in the studied hearts. The results are presented as mean ± 95% confidence intervals. Values presenting significant differences are marked with asterisk (* or **). DWTR: arterial diameter-to-wall thickness ratio; IVS: interventricular septum; LAR: arterial lumen-to-area ratio; LVW: left ventricular wall; RVW: right ventricular wall.

No differences were noted in the percentage of fibrosis in all three groups in the left ventricular wall, right ventricular wall and interventricular septum. The arterial DWTR and LAR were decreased in the left ventricular wall in both HCM and FHT groups as compared to controls, while in the interventricular septum DWTR was decreased only in the FHT group as compared to controls, and LAR was decreased in both FHT and HCM groups as compared to controls. In the right ventricular wall, LAR was decreased only in the FHT group as compared to the control group (Table 5, Figures 4 and 5).

## Discussion

Our study showed that although cats with hyperthyroidism present with similar features of myocardial degeneration and cardiomyocyte hypertrophy as the HCM cats, the macroscopic cardiac hypertrophy in the FHT animals is less prominent.

Young et al. (2022) showed that cats with hyperthyroidism tend to have a higher vertebral heart score on radiographic examination as compared to healthy animals, although the clinical relevance of this finding was considered unclear. In our study, the HCM cats showed significantly greater heart mass and larger heart diameter (both height and width) than healthy cats, while in the FHT group only the cardiac width was enlarged. Simultaneously, the thickness of the ventricular walls and the surface of the heart, of the left ventricle and of the left ventricular wall in cross section show no significant difference between the FHT and control groups with significantly higher values in the HCM group as compared to the other groups. No difference in body weight between the studied groups points to actual cardiomegaly and cardiac hypertrophy in the HCM cats and less prominent changes in hyperthyroid animals.

The reports on echocardiographic examination of cats with FHT report that left ventricular hypertrophy and left ventricular and left atrial dilation are less prominent than in cats with HCM (Liu et al. 1984; Moise et al. 1986; Bond et al. 1988). Similar lesions were visualised in experimental hyperthyroidism in pigs (Noszczyk-Nowak 2007; Noszczyk-Nowak et al. 2007). This is consistent with the results obtained in our study. In the left ventricle of the FHT cats, hypertrophy of the free wall is more common than that of the interventricular septum, while in HCM, septal hypertrophy is reported more often (Bond et al. 1988; Fox et al. 1995). In our study, the majority of the examined cats with cardiac hypertrophy showed a symmetrical pattern of changes. Nonetheless, a relatively small number of cats with wall hypertrophy in the FHT group does not allow drawing further conclusions on the observed pattern of hypertrophy. Moreover, we found no significant hypertrophy of either LVW, RVW or IVS in the FHT group, while in the HCM group, wall thickness was significantly larger than in the control group, which is consistent with data present in the literature (Kershaw et al. 2012; Biasato et al. 2015). The atrial appendages diameters were larger in the HCM group than in the control cats, while only the left atrial appendage width was greater in the FHT group as compared to controls. As mentioned previously, left atrial dilation is more severe in HCM than in hyperthyroidism (Liu et al. 1984; Moise et al. 1986; Bond et al. 1988). On the contrary, Biasato et al. (2015) showed no difference in the size of left atrial appendage between the HCM and healthy cats. We found no such study regarding feline hyperthyroidism.

As reported in the literature (Bond et al. 1988; Carney et al. 2016; Kittleson and Côté 2021), the changes in the hearts of hyperthyroid cats may resolve after treatment, explaining the normal values of LVW, IVS and RVW thickness obtained in our study. The majority of previous papers describe measurements taken during an echocardiographic examination. The post-mortem dimensions correspond directly with neither end-diastolic nor end-systolic measurements. While in humans (Prakash and Umali 1984), the necropsy left ventricular wall thickness measurements do not differ significantly from the echocardiographic systolic left ventricular wall thickness measurements, we found no such study on feline patients. Therefore, the obtained post-mortem results should not be extrapolated to clinical results.

The histological appearance of the hypertrophied hearts due to HCM has been previously described (Kershaw et al. 2012; Biasato et al. 2015). In our research, the histological changes of the HCM myocardium are consistent with the literature. The study conducted on cats with hyperthyroidism and signs of left ventricular hypertrophy (Liu et al. 1984) report several histological changes including: nuclear enlargement and hyperchromasia, interstitial fibrosis, endocardial fibroplasia, fibrosis of atrioventricular node, and marked disorganisation of cardiac muscle cells (disarray). Similar changes were noted in the left ventricle of a hyperthyroid rat model (Freitas et al. 2013). Interestingly, in our study, the severe histological changes were present in the FHT group despite the lack of cardiomegaly or gross cardiac hypertrophy. The myocardium of the FHT cats showed severe degeneration (in specimens obtained from all ventricular walls: the left ventricular wall, right ventricular wall and interventricular septum) and cardiomyocyte hypertrophy (in the interventricular septum and right ventricular wall specimens). The severe changes in the myocardium were visible, although the gross morphometric analysis of the ventricular wall thickness and surface did not show differences from the control group. Moreover, significant disarray was not observed in the FHT group as it was visible in the HCM group. In a rat model of hyperthyroidism, significant fibrosis was observed in the left ventricular specimens as compared to the control group (Freitas et al. 2013). In our study, the percentage of fibrosis in either of the specimens did not differ significantly between the groups.

In a rat model of hyperthyroidism, myocardial capillary rarefaction was noted (Freitas et al. 2013), pointing to an association between the elevated levels of thyroid hormones and cardiac microcirculation disorders. Nonetheless, myocardial coronary arteries in FHT have not previously been described in the literature. In dogs with experimentally induced hyperthyroidism, the smooth muscle of the coronary arteries was markedly hypertrophied with no signs of cardiac wall thickening after 14 days of triiodothyronine injections (Hoey et al. 1991). In our study, the alterations in arterial morphology were more prominent in the FHT group than in the HCM group, showing significant ­narrowing of the arterial lumen in all ventricular walls. In humans, hyperthyroidism (including the subclinical form) is combined with a higher risk of death from coronary heart disease, especially in elderly patients (Fadel et al. 2000; Biondi 2021). Moreover, in humans with Graves’ disease increased systemic arterial stiffness was reported (Bodlaj et al. 2007). This may point to a role of structural changes of the arteries (including both systemic and cardiac vasculature) in the pathogenesis of secondary changes in hyperthyroidism in cats and humans. In cats, antihypertensive drugs are used as part of the therapeutic plan, with amlodipine besylate being the first-choice antihypertensive treatment in cats (Acierno et al. 2018).

There are some limitations of our study. The number of the cases studied did not allow us to draw further conclusions on the impact on the observed changes of: the initial severity of hyperthyroidism, a dose and duration of the treatment, the effect of the treatment, the survival time and the clinical signs of cardiac involvement and/or heart failure in the FHT group. Secondly, the detailed results of echocardiographic examination were not available in all cases. Moreover, cats in the FHT group were treated pharmacologically, which may lead to fluctuations of the thyroid hormone levels, which in turn may affect the final outcome. Therefore, further studies involving larger groups of cats together with detailed clinical information should be conducted.

## Conclusions

Our study showed that hyperthyroidism in cats is related to lesser gross cardiac hypertrophy and cardiomegaly than hypertrophic cardiomyopathy. Despite normal values of wall thickness, relevant cardiac degeneration, cardiomyocyte hypertrophy and intramural arterial alterations were noted in hyperthyroid cats. The structural changes involved not only the previously described left ventricular wall and interventricular septum but also the right ventricular wall, leading to generalised impairment of the cardiac structure. The vascular alterations were more severe in hyperthyroid cats as compared to animals with hypertrophic cardiomyopathy, regardless of similar changes in the cardiomyocyte structure in these two groups.

Further studies are required to better understand the myocardial vasculature response to hyperthyroidism. Broadening our knowledge may lead to new treatment strategies that can improve the outcome in both feline and human patients with thyroid diseases.

## Supplementary Material

Supplemental Material

## Data Availability

The research data are available at the main author after reasonable request
