# Supplementary material for: Comparative cardiac macroscopic and microscopic study in cats with hyperthyroidism vs. cats with hypertrophic cardiomyopathy
Source: Vet Q. 2023 Jul 18;43(1):1–11. doi: 10.1080/01652176.2023.2234436 (PMC10355689; doi:10.1080/01652176.2023.2234436)
Supplement: Supplemental Material [file TVEQ_A_2234436_SM2467.docx]

Supplementary Material

The type and duration of treatment in animals diagnosed with hyperthyroidism (FHT) or hypertrophic cardiomyopathy (HCM). The animals enrolled as a control group have not received any treatment prior to euthanasia.

| Number of animal | Group | Drug | Dose | Duration |
| --- | --- | --- | --- | --- |
| 1 | FHT | thiamazole | 2.5 mg/cat BID | 2 years |
| 2 | FHT | thiamazole | 2.5 mg/cat BID | > 1 year^2^ |
| 3 | FHT | n/a^1^ |  |  |
| 4 | FHT | n/a^1^ |  |  |
| 5 | FHT | thiamazole | 2.5 mg/cat BID | 3 months |
| 6 | FHT | thiamazole | 2.5-5 mg/cat BID | 9 months |
| 7 | FHT | thiamazole | 2.5 mg/cat BID | 1 year |
| 8 | FHT | thiamazole | 2.5 mg/cat BID | 6 months |
|  |  | torasemide | 0.2-0.3 mg/kg SID | 1 week |
|  |  | clopidogrel | 18.75 mg/cat SID | 1 week |
| 9 | FHT | thiamazole | 2.5 mg/cat BID | 2 weeks |
| 10 | FHT | thiamazole | 2.5 mg/cat BID | 1.5 year |
|  |  | torasemide | 0.2-0.4 mg/kg SID | 3 weeks |
|  |  | clopidogrel | 18.75 mg/cat SID | 3 weeks |
| 11 | FHT | thiamazole | 2.5 mg/cat BID | >1 year^2^ |
| 12 | FHT | n/a^1^ |  |  |
| 13 | FHT | thiamazole | 2.5-5 mg/cat BID | 2 years |
| 14 | FHT | thiamazole | 2.5-5 mg / cat BID | >3 years^2^ |
| 15 | FHT | n/a^1^ |  |  |
| 16 | FHT | thiamazole | 2.5 mg/cat BID | 1 year |
| 17 | FHT | n/a^1^ |  |  |
| 18 | HCM | n/a^1^ |  |  |
| 19 | HCM | torasemide | 0.2-0.5 mg/kg SID | 4 years |
|  |  | clopidogrel | 18.75 mg/cat SID | 4 years |
|  |  | atenolol | 6.25 mg/cat BID | 4 years |
| 20 | HCM | n/a^1^ |  |  |
| 21 | HCM | torasemide | 0.3-0.4 mg/kg SID | 1 month |
|  |  | clopidogrel | 18.75 mg/cat SID | 1 month |
| 22 | HCM | toasemide | 0.2-0.3 mg/kg SID | 1.5 year |
|  |  | clopidogrel | 18.75 mg/cat SID | 1.5 year |
|  |  | atenolol | 6.25-12.5 mg/cat BID | 1.5 year |
| 23 | HCM | spironolactone | 1 mg/kg SID | 6 months |
| 24 | HCM | n/a^1^ |  |  |
| 25 | HCM | n/a^1^ |  |  |
| 26 | HCM | torasemide | 0.2-0.4 mg/kg SID | 2 years |
|  |  | clopidogrel | 18.75 mg/cat SID | 2 years |
|  |  | atenolol | 6.25-12.5 mg/cat BID | 2 years |
| 27 | HCM | n/a^1^ |  |  |
| 28 | HCM | n/a^1^ |  |  |
| 29 | HCM | n/a^1^ |  |  |
| 30 | HCM | n/a^1^ |  |  |

^1^ the animal was euthanised directly after the diagnosis due to poor clinical condition and prognosis; no treatment was applied before the euthanasia

^2^ prior medical history was not available in case of the animal; therefore, a direct duration of therapy cannot be determined
